# Targets to Search for New Pharmacological Treatment in Idiopathic Parkinson’s Disease According to the Single-Neuron Degeneration Model

**DOI:** 10.3390/biom14060673

**Published:** 2024-06-08

**Authors:** Sandro Huenchuguala, Juan Segura-Aguilar

**Affiliations:** 1 Escuela de Tecnología Médica, Facultad de Salud, Universidad Santo Tomás, Santiago 8370003, Chile; shuenchuguala@santotomas.cl; 2 Molecular & Clinical Pharmacology, ICBM, Faculty of Medicine, University of Chile, Santiago 8330111, Chile

**Keywords:** dopamine, VMAT2, neuromelanin, neurodegeneration, neuroprotection, Parkinson’s disease, pramipexole, nicotine, KEAP1/NRF2

## Abstract

One of the biggest problems in the treatment of idiopathic Parkinson’s disease is the lack of new drugs that slow its progression. L-Dopa remains the star drug in the treatment of this disease, although it induces severe side effects. The failure of clinical studies with new drugs depends on the use of preclinical models based on neurotoxins that do not represent what happens in the disease since they induce rapid and expansive neurodegeneration. We have recently proposed a single-neuron degeneration model for idiopathic Parkinson’s disease that requires years to accumulate enough lost neurons for the onset of motor symptoms. This single-neuron degeneration model is based on the excessive formation of aminochrome during neuromelanin synthesis that surpass the neuroprotective action of the enzymes DT-diaphorase and glutathione transferase M2-2, which prevent the neurotoxic effects of aminochrome. Although the neurotoxic effects of aminochrome do not have an expansive effect, a stereotaxic injection of this endogenous neurotoxin cannot be used to generate a preclinical model in an animal. Therefore, the aim of this review is to evaluate the strategies for pharmacologically increasing the expression of DT diaphorase and GSTM2-2 and molecules that induce the expression of vesicular monoamine transporter 2, such as pramipexole.

## 1. Parkinson’s Disease

Parkinson’s disease is a neurodegenerative disease that affects the control of the motor system. Although intensive research has been carried out for decades to decipher the molecular mechanism that triggers this disease, it is still not clear what triggers the degeneration of the neuromelanin-containing dopaminergic neurons of the nigrostriatal system.

The discovery in 1960 that low dopamine levels in Parkinson’s disease are a product of the loss of dopaminergic neurons that contain neuromelanin has been one of the most important discoveries in understanding the molecular mechanisms of this disease. However, although several mechanisms related to the neurodegeneration of the nigrostriatal system have been identified, such as mitochondrial dysfunction, oxidative stress, dysfunction of both proteasomal and lysosomal protein degradation systems, endoplasmic reticulum stress, neuroinflammation, and the formation of neurotoxic oligomers of alpha-synuclein, it is not yet known what triggers these mechanisms [1,2,3,4,5,6,7].

A great advance in the research into molecular mechanisms has been the discovery of several mutations associated with familial Parkinson’s disease, since it has made it possible to reveal certain proteins that play a role in the development of Parkinson’s disease symptoms. The first of these proteins was a mutation in the alpha-synuclein gene that induces the formation of neurotoxic oligomers. Other mutations in genes that are involved in mitochondrial dysfunction include parkin, PTEN-induced putative kinase 1, and Protein/nucleic acid deglycase 1 [8]. Mutations in the leucine-rich repeat kinase 2 gene that would be involved in the control of membrane trafficking and lysosomal function have been reported [9]. Mutations in the ubiquitin carboxy-terminal hydrolase-L1, ATPase Cation Transporting 13A2, and other genes have also been reported to be associated with familial Parkinson’s disease [10]. The discovery of these genes associated with familial Parkinson’s disease has been a great contribution to molecular studies of the disease. The mutation of alpha-synuclein that induces its aggregation into neurotoxic oligomers resulting in lysosomal, mitochondrial, and endosomal dysfunction has had an enormous impact on basic research. The discovery of the ability of alpha-synuclein aggregates to spread between the gut, brainstem, and higher brain regions has allowed some researchers to suggest the stage hypothesis of Parkinson’s disease [11]. However, it must be remembered that familial Parkinson’s disease only represents 5–10% of the cases of this disease and it is a mistake to think that preclinical models with mutations of these genes will represent what happens in patients with idiopathic Parkinson’s disease. However, several preclinical models have been developed with animals that express mutations of some genes associated with familial Parkinson’s disease [12,13,14]. Overexpression of the human alpha-synuclein gene in rats induces an alteration in the gut microbiota [15].

There are many researchers who consider that environmental exposure plays an important role in the degenerative process of the nigrostriatal system that induces the loss of neuromelanin-containing dopaminergic neurons. Others think that exposure to heavy metals, solvents, pesticides, and environmental toxins could be partly responsible for the rapid growth in Parkinson’s disease [16]. A study on the possible role of exposure to local traffic-related air pollution in central California, USA, which included 761 patients with Parkinson’s disease and 910 healthy controls, concluded that exposure to local traffic-related air pollution is associated with an increased risks of developing Parkinson’s disease [17]. Exposure of workers in manganese mines was reported more than 60 years ago. Exposure to manganese is not only limited to manganese mines but also to workers who work in welding where they are exposed to the fumes that develop during this activity [18]. However, in subjects with Parkinsonism induced by metals such as manganese, copper, or pesticides such as paraquat, atypical Parkinsonism with early onset is induced in young workers [19,20]. This group of people exposed to pollutants who develop Parkinsonism cannot be included in the group of idiopathic Parkinson’s disease patients and constitute a special group that constitute approximately 20% of the total Parkinsonian individuals.

The possibility that environmental factors may play a role in triggering the mechanisms involved in the degenerative process and loss of neuromelanin-containing dopaminergic neurons of the nigrostriatal system in idiopathic Parkinson’s disease is questionable. The best evidence that exogenous factors or neurotoxins do not play a role in idiopathic Parkinson’s disease is from drug addicts exposed to 1-methyl-4-phenyl-1,2,3,6-tetrahydropyridine (MPTP). Subjects who consumed drugs contaminated with MPTP developed severe Parkinsonism in just three days [21] which contrasts with the extremely slow generative and propagation process of idiopathic Parkinson’s disease, which takes many years.

The existence of premotor symptoms has been reported as olfactory dysfunctions, depression, insomnia, anxiety, rapid eye movement sleep behavior, constipation, and cognitive decline [22]. It has been proposed that Lewy bodies and Lewy neurites with alpha-synuclein immunoreactive deposits expand from regions such as the anterior olfactory nucleus to other regions, until they affect the substantia nigra where motor symptoms are generated [23]. However, this hypothesis of stages of the disease that progress from region to region of the brain of the patient with Parkinson’s disease is controversial since it would be valid for patients with an early onset of the disease but not for patients with a late onset such as the idiopathic form of the disease [24]. Although there may be premotor symptoms, they ultimately all come together in the loss of dopaminergic neurons that contain neuromelanin.

The extremely rapid effect of MPTP-Induced Parkinsonism in individuals exposed to this drug suggests that the neurotoxin that triggers neurodegeneration of the nigrostriatal system in idiopathic Parkinson’s disease cannot be of exogenous origin since it will have a massive, expansive, and rapid neurodegeneration [21].

Exposure to manganese, copper, and 3,4-methylenedioxymethamphetamine also leads to the development of early-onset Parkinsonism [20,25,26]. On the contrary, to trigger an extremely slow degenerative process that takes years, such as in idiopathic Parkinson disease, the neurotoxin that triggers this neurodegeneration is presumably of endogenous origin and does not have an expansive character. This endogenous neurotoxin is probably formed inside the dopaminergic neurons that contain neuromelanin. Possible neurotoxins generated within dopaminergic neurons are neurotoxic oligomers of alpha-synuclein, 3,4-dihydroxyphenylacetaldehyde (DOPAL) and aminochrome that are formed during neuromelanin synthesis.

Alpha-synuclein is normally found in its monomeric state but under certain circumstances it can be aggregated to fibrils that accumulate in Lewy bodies and Lewy neurites, which are not exclusive to the nigrostriatal system, but this aggregation occurs in other brain regions that are expanding from region to region [27,28]. Alpha-synuclein has the ability to spread from neuron to neuron through its secretion and subsequent uptake of the receiving neuron [29,30]. Seed spread of alpha-synuclein fibrils has been observed in different brain regions and serum of patients [31,32,33]. The internalization of alpha-synuclein released is an endosome-lysosome mechanism [34]. A stage of different levels of development of Parkinson’s disease based on the spread of Lewy bodies has been proposed. This disease stage helps explain the appearance of premotor symptoms [23]. However, the role of alpha-synuclein aggregation to fibrils and its formation of deposits in Lewis bodies that spread across brain regions has been questioned. It has been suggested that the Braak stages are not valid for patients with late onset such as patients with idiopathic Parkinson’s disease, but are valid for patients with early onset of the disease and those with long-lasting motor symptoms [24].

Another argument against the role of Lewy bodies in Parkinson’s disease pathology is the absence of Lewy bodies in patients with familial Parkinson’s disease associated with mutations in the Parkin and leucine-rich repeat kinase 2 genes [35,36,37,38]. Lewy bodies loaded with alpha-synuclein are observed in postmortem tissues from patients with Parkinson’s disease, which correspond to tissues that have survived the degenerative process for years. If alpha-synuclein deposits in Lewy bodies were neurotoxic, they could not be observed in postmortem tissue from patients with Parkinson’s disease [39]. Another point against the neurotoxic role of Lewy bodies loaded with alpha-synuclein deposits is the propagative nature of these Lewy bodies. The spread of alpha-synuclein fibrils deposited in Lewy bodies through exosomes [40] occurs intracellularly to neighboring neurons and subsequently to other regions. This propagative mode of alpha-synuclein fibril transfer to other neurons should imply a rapid progression of the neurodegenerative process and disease progression because the propagation of these fibrils does not involve a single fibril but a large number. In addition, it has been proposed that the accumulation of alpha-synuclein fibrils in Lewy bodies could actually be a neuroprotective mechanism [41].

Monomeric alpha-synuclein is also aggregated to oligomers that are considered the species responsible for the neurotoxic action of this protein [42,43]. Alpha-synuclein oligomers induce synaptic impairment, endoplasmic reticulum stress, mitochondrial dysfunction, loss of regulation of proteostasis, neuroinflammation, cell apoptosis, lysosomal dysfunction, oxidative stress, and autophagy impairment [27,44,45,46,47,48]. However, the propagative nature of alpha-synuclein [49] will imply rapid neurodegeneration of the nigrostriatal system when neurotoxic oligomers are formed, which is the opposite of what occurs in the disease. Mutations in the alpha-synuclein gene induce the formation of neurotoxic alpha-synuclein oligomers in familial Parkinson’s disease that are transmitted to neighboring neurons through exosomes [49,50,51,52]. Alpha-synuclein alone does not aggregate into neurotoxic oligomers and the question is what induces the aggregation of alpha-synuclein to neurotoxic oligomers in neuromelanin-containing dopaminergic neurons in the nigrostriatal system in idiopathic Parkinson’s disease. In in vitro experiments the formation of oligomers was reported in the presence of iron, copper, manganese, DOPAL, or rotenone [53,54,55,56,57]. However, the massive degeneration that these metals can generate that induces early-onset Parkinsonism in young workers [20,58] does not coincide with the extremely slow degenerative process that occurs in idiopathic Parkinson’s disease, which takes years. An experiment in mice revealed an increase in alpha-synuclein oligomers and neurodegeneration by increasing dopamine and alpha-synuclein levels [59]. Alpha-synuclein aggregates were observed during dopamine oxidation catalyzed by aminochrome and 5,6-indolequinone [60,61]. Aminochrome forms neurotoxic oligomers in cell culture when the enzyme DT-diaphorase is silenced with siRNA [62].

Aminochrome is an endogenous neurotoxin that is an intermediate formed in the synthesis of neuromelanin inside of the neurons lost in Parkinson’s disease. Neuromelanin is synthesized from the oxidation of the catechol dopamine group to three ortho-quinones (dopamine --> dopamine ortho-quinone --> aminochrome --> 5,6-indolequinone --> neuromelanin) [63,64]. These ortho-quinones are potentially neurotoxic but aminochrome is the most stable and neurotoxic [65,66]. Aminochrome induces oxidative stress, mitochondrial dysfunction, formation of neurotoxic alpha-synuclein oligomers, dysfunction of both lysosomal and proteasomal protein degradation systems, neuroinflammation, and endoplasmic reticulum stress [67,68,69,70,71,72].

Another neurotoxin that can be formed in dopaminergic neurons is DOPAL, which is the product of the oxidative deamination of dopamine catalyzed by monoamine oxidase [73]. DOPAL is converted to 3,4-dihydroxyphenylacetic acid catalyzed by the enzyme aldehyde dehydrogenase-1. DOPAL can form adducts with alpha-synuclein, generating the formation of oligomers and their accumulation that induce neurodegeneration [55]. The addition of DOPAL and A53T alpha-synuclein to glial cells demonstrated that glia cells can take up DOPAL and increase alpha-synuclein oligomerization intracellularly [74]. DOPAL-induced alpha-synuclein oligomerization increases in the presence of divalent metals such as Cu^2+^, Fe^2+^, and Mn^2+^ [75]. A study with astrocytes showed that DOPAL induces apoptosis and oxidative and nitrative stress, and lowers mitochondrial function. An experiment performed with postmortem tissue from patients with Parkinson’s disease revealed a low expression of the enzyme aldehyde dehydrogenase-1 [76]. A low expression of this enzyme would imply an accumulation of DOPAL that can be oxidized to ortho-quinones and have neurotoxic effects [73,76,77]. However, this low expression was observed in postmortem tissue from patients with late-onset Parkinson’s disease who survived the neurodegenerative process, which raises questions as to its role as an endogenous neurotoxin that triggers the neurodegenerative process.

The aim of this review is to propose a different point of view on how to approach the search for new drugs to halt or reduce the progression of idiopathic Parkinson’s disease, based on a new concept to interpret the degenerative process of neuromelanin-containing dopaminergic neurons as a single-neuron degeneration model [78] (Table 1).

## 2. Dopamine Metabolism

Dopamine is synthesized in the cytosol of dopaminergic neurons from the amino acid L-tyrosine that is converted into L-3,4-dihydroxyphenylalanine (L-Dopa) in a reaction catalyzed by the enzyme tyrosine hydroxylase where a hydroxyl group is introduced into position 3 of tyrosine forming a catechol structure [79]. L-Dopa is subsequently decarboxylated forming dopamine in a reaction catalyzed by the enzyme aromatic L-amino acid decarboxylase [80]. The objective of dopamine synthesis is its accumulation in monoaminergic neurotransmission vesicles through vesicular monoamine transporter-2 (VMAT2), which catalyzes its uptake from the cytosol into the interior of these vesicles [81].

Free dopamine in the cytosol can be degraded through its oxidative deamination catalyzed by the enzyme monoamine oxidase. Alternatively, free dopamine in the cytosol can be oxidized to neuromelanin. The catechol group of dopamine is oxidized to form three ortho-quinones sequentially, namely dopamine ortho-quinone, aminochrome, and 5,6-indolequinone, which finally polymerize to form neuromelanin [65,79].

The synthesis of neuromelanin is a normal and harmless pathway since, in healthy older adults, the neuromelanin-containing dopaminergic neurons are intact at the time of death [82]. However, in older adults with Parkinson’s disease, the majority of neuromelanin-containing dopaminergic neurons have been lost [83,84]. The reason that neuromelanin-containing dopaminergic neurons are lost in the substantia nigra of patients with Parkinson’s disease depends on the neurotoxic action of transient ortho-quinones that are formed during neuromelanin synthesis. Aminochrome is the most stable and neurotoxic transient ortho-quinone since: (i) it can be reduced with one electron by flavoenzymes that transfer an electron to a leukoaminochrome o-semiquinone radical, which is extremely reactive with oxygen [85]. Autoxidation of the leukoaminochrome o-semiquinone radical generates redox cycling between aminochrome and leukoaminochrome o-semiquinone that reduces dioxygen to superoxide. This redox cycling implies that the dioxygen that is needed to complete the mitochondrial electron transfer that is ultimately required for oxidative phosphorylation of ADP to ATP is depleted. Additionally, this redox cycling also depletes NADH which is used in the mitochondrial electron transport chain. Finally, this redox cycling generates oxidative stress and ATP depletion that is required, among other things, for the neurotransmission of dopamine from monoaminergic vesicles; and (ii) aminochrome is also capable of forming adducts with proteins such as alpha-synuclein, actin, α and β-tubulin, mitochondrial complex 1, ATP13A, and other proteins [62,86,87]. The neurotoxic effects of aminochrome induce oxidative stress, neuroinflammation, formation of neurotoxic alpha-synuclein oligomers, mitochondrial dysfunction, endoplasmic reticulum stress, and dysfunction of both lysosomal and proteasomal protein degradation systems [67,68,69,70,71,72].

Aminochrome is a transient metabolite that in in vitro experiments monitored with NMR has been determined to be stable 40 min before beginning its conversion to 5,6-indolequinone, which polymerizes to neuromelanin [66]. However, in the cytosol of a neuron that is full of proteins, enzymes, lipids, and other biomolecules, the stability of aminochrome is much lower since it is either reduced by flavoenzymes or forms adducts with proteins, which prevents this endogenous neurotoxin from having an expansion that affects neighboring neurons. This implies that the neurotoxic effects of aminochrome only affect a single neuron. This single-neuron degeneration model could explain why the loss of neuromelanin-containing dopaminergic neurons in a patient with idiopathic Parkinson’s disease is extremely slow, taking years before the onset of motor symptoms and also later during the progression of the disease [78] (Table 2).

## 3. Neuroprotection against Aminochrome Neurotoxicity

The question is how neuromelanin synthesis can be a normal and harmless process if it requires the formation of the endogenous neurotoxin aminochrome. This can be explained by the existence of the enzyme DT-diaphorase that reduces two-electron aminochrome to leukoaminochrome, preventing the reduction of one-electron aminochrome to a leukoaminochrome o-semiquinone radical catalyzed by flavoenzymes that reduce one-electron quinones [88,89]. DT-diaphorase prevents the aminochrome-induced death of dopaminergic neurons, mitochondrial dysfunction, oxidative stress, lysosomal dysfunction, disruption of cytoskeletal architecture, dysfunction of protein degradation of the proteasomal system, and autophagy [65,68,90].

In 1997 we began a scientific collaboration with Professor Bengt Mannervik to study the ability of glutathione transferases to conjugate aminochrome. Interestingly, human glutathione transferase M2-2 was the most active isoenzyme within these isoenzymes and its conjugate 4-S-glutathionyl-5,6-dihydroxyindoline is resistant to biological oxidative agents such as dioxygen, superoxide, and hydrogen peroxide [91]. Glutathione transferase M2-2 conjugates not only aminochrome but also its precursor dopamine ortho-quinone to 5-glutathioneyldopamine, which is degraded to 5-cysteinyldopamine [92]. Interestingly, 5-cysteinyldopamine has been detected in neuromelanin and human cerebrospinal fluid, suggesting that it is a final reaction in which a stable product is produced that is eliminated from neuromelanin-containing dopaminergic neurons into the cerebrospinal fluid and accumulated in neuromelanin [93,94]. Glutathione transferase M2-2 is not expressed in neuromelanin-containing dopaminergic neurons where the aminochrome triggers the degeneration of these neurons. Astrocytes can take up dopamine released during neurotransmission. Dopamine within astrocytes can be oxidized to aminochrome, where glutathione transferase M2-2 can conjugate both aminochrome and its precursor dopamine ortho-quinone. However, it has been reported that astrocytes secrete exosomes loaded with glutathione transferase M2-2 that penetrate dopaminergic neurons, discharging this enzyme into their cytosol to increase the protection of these neurons against the neurotoxic effects of aminochrome [90,95,96,97] (Table 3).

## 4. Prevention of Dopamine Oxidation-Dependent Neurotoxicity

One of the fundamental events in the progression of idiopathic Parkinson’s disease is the appearance of motor symptoms when 60% of neuromelanin-containing dopaminergic neurons are lost [98]. However, it has been proposed that the onset of the disease is observed when 50–60% of the dopaminergic terminals of the striatum have been lost, which would correspond to a 30% loss of dopaminergic neurons of the substantia nigra [99]. The speed of the degenerative process of neuromelanin-containing dopaminergic neurons in the nigrostriatal system is extremely slow and lasts for many years [78]. This suggests that this degenerative process is not expansive in nature and that the neurotoxin that triggers it seems to be of endogenous origin. Therefore, the prevention of dopamine oxidation to aminochrome is a potential way to inhibit the loss of neuromelanin-containing dopaminergic neurons in idiopathic Parkinson’s disease, if we consider that the oxidation of dopamine to aminochrome plays an essential role in the degenerative process of neuromelanin-containing dopaminergic neurons [100].

The oxidation of dopamine to neuromelanin depends on the existence of free dopamine in the cytosol and the presence of metals or enzymes with peroxidase activity. One of the possible sources of free dopamine is its synthesis from the amino acid L-tyrosine, which requires the action of two enzymes (tyrosine hydrolase and aromatic enzyme L-amino acid decarboxylase). Subsequently, vesicular monoamine transporter 2 (VMAT-2), which is expressed in monoaminergic neurotransmission vesicles, transports the newly synthesized dopamine into the vesicles. Inside the monoaminergic neurotransmission vesicles, dopamine can accumulate in high concentrations without the risk of oxidation since these vesicles have a slightly acidic pH inside [101]. These vesicles have an H+-ATPase that pumps protons into these vesicles, generating a slightly acidic pH inside them [81].

Interestingly, the enzymes tyrosine hydrolase and aromatic enzyme L-amino acid decarboxylase form a type of complex with VMAT-2 that prevents the existence of free dopamine since the newly synthesized L-dopa is immediately converted into dopamine that is transported to the monoaminergic vesicles of neurotransmission catalyzed by VMAT-2 [102]. The other source of free dopamine in the cytosol of neuromelanin-containing dopaminergic neurons is the reuptake of dopamine released during neurotransmission via dopamine transporters. However, the dopamine transporter, VMAT-2 and synaptogyrin-3 also form a type of complex that prevents the dopamine reuptake by the dopamine transporter from being released directly into the cytosol [103]. Therefore VMAT-2 plays a key role in preventing the oxidation of dopamine to neuromelanin in the cytosol of dopaminergic neurons.

The level of VMAT-2 expression may play a fundamental role in preventing the oxidation of dopamine to neuromelanin that generates three potentially neurotoxic ortho-quinones. There is an inverse relationship between neuromelanin levels and VMAT-2 expression which is based on the fact that the major accumulation of neuromelanin occurs in the substantia nigra, which has less VMAT-2 expression compared to the midbrain dopaminergic neurons of VTA that have less neuromelanin formation despite producing more dopamine and a higher expression of VMAT-2 [104,105]. The possibility that the degeneration of axons [99] may depend on the oxidation of dopamine to aminochrome due to the leak of dopamine from the monoaminergic vesicles located in the dopaminergic terminals located in the striatum does not seem to be feasible because the presence of neuromelanin has not been observed in the striatum (Table 4).

## 5. Clinical Studies in Parkinson’s Disease

One of the biggest concerns in Parkinson’s disease research is the failure of all clinical studies of drugs that aimed to modify the course of the disease (isradipine, coenzyme Q10, TCH346, mitoquinone, nilotinib, zonisamide, deferiprone, prasinezumab, and cinpanemab) or regenerate dopaminergic neurons (neurturin analogue of GDNF) [106]. All these clinical studies were based on successful preclinical studies that used exogenous neurotoxins such as MPTP or 6-hydroxydopamine, which induce a rapid, massive, and propagative degenerative process [107]. Preclinical studies with coenzyme Q10 were successful [108,109,110] but clinical studies did not show a benefit for patients with Parkinson’s disease [111]. Mito-Q(10), a modified coenzyme Q10 analogue, showed a clear neuroprotective effect in MPTP and a 6-hydroxydopamine preclinical model [112,113,114] but in clinical studies they did not show neuroprotective effects in patients with Parkinson’s disease [115]. Neuroprotective effects of urate were demonstrated in preclinical models based on 6-hydroxydopamine [116,117,118]. However, a clinical study failed to show benefits in patients with Parkinson’s disease [119]. One of the possible explanations for the failure of these clinical studies is that the degenerative process of idiopathic Parkinson’s disease is extremely slow. The evaluation of patients with MDS-UPDRS is unable to detect progress as a result of the therapeutic action of the drugs used in these clinical studies because the progress of the neurogenerative process is so slow. Recently, it has been published that the number of dopaminergic neurons of the substantia nigra considering both hemispheres varies between 800,000 and 1,000,000 dopaminergic neurons, which implies that when the motor symptoms appear in the disease, only between 320,000 to 400,000 dopaminergic neurons remain, after 60% of those neurons have disappeared [120]. As the degenerative process of the nigrostriatal system in idiopathic Parkinson’s disease is extremely slow, it is possible that the positive therapeutic effect observed in these preclinical studies with exogenous neurotoxins is impossible to determine in clinical studies because the speed of the degenerative process in the disease is extremely slow. Recently, the single-neuron degeneration model has been proposed, where the degenerative process is induced by the endogenous neurotoxin aminochrome that induces non-propagative neurodegeneration. The loss of a single neuromelanin-containing dopaminergic neuron accumulates over years until reaching a 60% loss when motor symptoms appear [78] (Table 5).

## 6. VMAT-2 as a Target to Develop New Drugs for Parkinson’s Disease

It is urgent to search for new therapeutic targets for Parkinson’s disease therapy, but the choice of the preclinical model is key to success. Based on the extreme slowness of the degenerative process and the progress of Parkinson’s disease, which takes years, we consider that the search for new targets with therapeutic effects on the disease should be based on a single-neuron degeneration model. The oxidation of dopamine to aminochrome during the synthesis of neuromelanin plays an essential role in the degenerative process of dopaminergic neurons containing neuromelanin in the nigrostriatal system. Therefore, the prevention of the oxidation of dopamine to aminochrome may be the most neuroprotective action to protect these neurons that are lost in Parkinson’s disease in the substantia nigra.

The role of VMAT-2 in preventing the oxidation of dopamine to neuromelanin has been described for many years [104,105]. However, the inhibition of VMAT-2 expression in the single-neuron degeneration model plays a key role since inhibiting the oxidation of dopamine to aminochrome does not require the overexpression of the neuroprotective enzymes DT-diaphorase and glutathione transferase M2-2 to prevent the neurotoxic effects of aminochrome [90]. The essential role of VMAT2 in preventing neurodegeneration of dopaminergic neurons containing neuromelanin-dependent dopamine oxidation has been demonstrated with the use of viral-mediated small-hairpin RNA interference of VMAT2. Loss of VMAT2 expression resulted in increased cytosolic dopamine concentration and subsequent degeneration of the nigrostriatal dopaminergic system. The addition of exogenous VMAT2 prevents the neurotoxic effects created by silencing the expression of this transporter [121].

**Figure 1:**
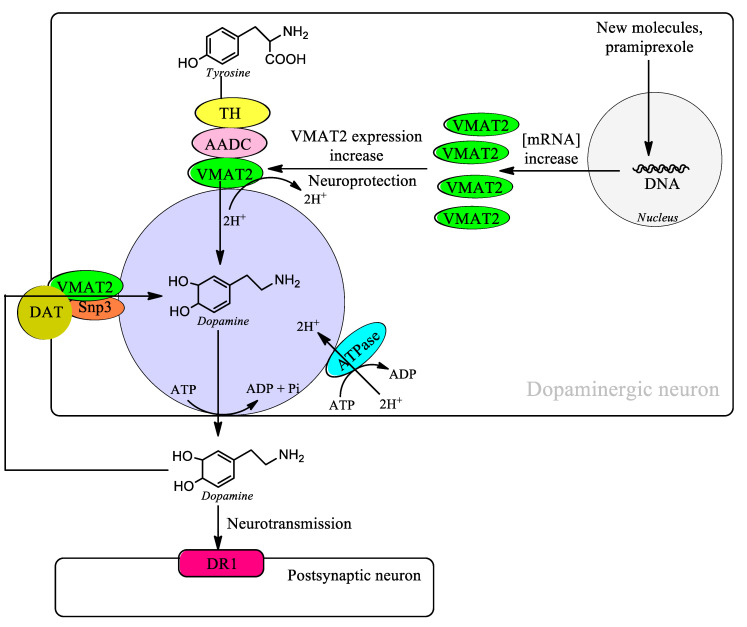
Increased expression of VMAT2 prevents the existence of free dopamine in the cytosol and the synthesis of aminochrome during neuromelanin synthesis.

Pramipexole is a dopamine agonist used in the therapy of Parkinson’s disease, and SPECT studies have demonstrated a neuroprotective effect in patients with Parkinson’s disease. Patients treated with the agonist ropinirole also showed a significant neuroprotective effect on nigrostriatal neurons [122]. The neuroprotective effects of pramipexole in patients with Parkinson’s disease are controversial since in a study with patients diagnosed within two years from an age range of 30 to 79 years, no significant differences were observed at 15 months [123]. However, this study included patients with early and late onset and did not focus solely on patients with idiopathic Parkinson’s disease. In a study carried out in the human neuroblastoma cell line SH-SY5Y, it has been shown that pramipexole induces the expression of VMAT2 mRNA levels, which suggests the neuroprotective effect observed in SPECT studies [124]. This suggests that the search for molecules that induce the expression of VMAT2 may be a target for the search for new neuroprotective molecules in the treatment of Parkinson’s disease that can change the course of the disease. The increase in the expression of VMAT2 implies a risk of the existence of free dopamine in the cytosol that can be oxidized to neuromelanin that requires the formation of aminochrome that can potentially be neurotoxic decreases. (Figure 1, Table 6).

VMAT2 is in the membrane of monoaminergic vesicles and catalyzes the transport of dopamine into the interior of monoaminergic vesicles where it is completely stable thanks to a slightly acidic environment. VMAT2 plays an essential role in preventing the existence of free dopamine in the cytosol and its oxidation to neuromelanin. Free dopamine in the cytosol can exist thanks to the synthesis of dopamine from the amino acid tyrosine and the reuptake of dopamine released during neurotransmission through the dopamine transporter (DAT). However, during dopamine synthesis VMAT forms a kind of complex with the enzymes tyrosine hydroxylase (TH) and aromatic L-amino acid decarboxylase (AADC) that prevents the existence of free dopamine in the cytosol. During the reuptake of dopamine through DAT after neurotransmission VMAT2 also forms a kind of complex with DAT and synaptogyrin-3 (Snp3) that prevents the existence of free dopamine in the cytosol.

## 7. Kelch-like ECH-Associated Protein 1/Nuclear Factor E2-Related Factor 2 (KEAP1/NRF2) Activation as a Target to Develop New Drugs for Parkinson’s Disease

The expression of antioxidant enzymes such as superoxide dismutase, glutathione peroxidase, glutathione transferase, catalase, heme oxygenase, and DT-diaphorase play an important role in neutralizing the effects of oxidative stress. Activation of the KEAP1/NRF2 signaling pathway allows NRF2 to activate the expression of antioxidant enzyme genes by binding to the antioxidant responsive element [125]. However, the activation of the KEAP2/NRF2 pathway in cancer cells can help develop resistance to antineoplastic drugs in which its mechanism of action is related to the generation of oxidative stress in cancer cells such as ovarian cancer or cervical and endometrial cancer [126,127]. In patients with preeclampsia, the activation of the KEAP1/NRF2 pathway has a protective effect to help neutralize oxidative stress and inflammation [128]. In other pathologies, the activation of the KEAP1/NRF2 pathway can have a protective effect, such as in celiac disease [129], ischemia/reperfusion [130], traumatic lung injury [131], nephrolithiasis [132], cardiovascular disease [133], and renal injury [134].

In the case of Parkinson’s disease, oxidative stress is one of the mechanisms involved in the degenerative process of neuromelanin-containing dopaminergic neurons [78]. Furthermore, the KEAP1/NRF2 signaling pathway induces the enzymes DT-diaphorase and glutathione transferase M2-2 [135,136,137], which prevent the neurotoxic effects of aminochrome that is formed during the synthesis of neuromelanin [78].

**Figure 2:**
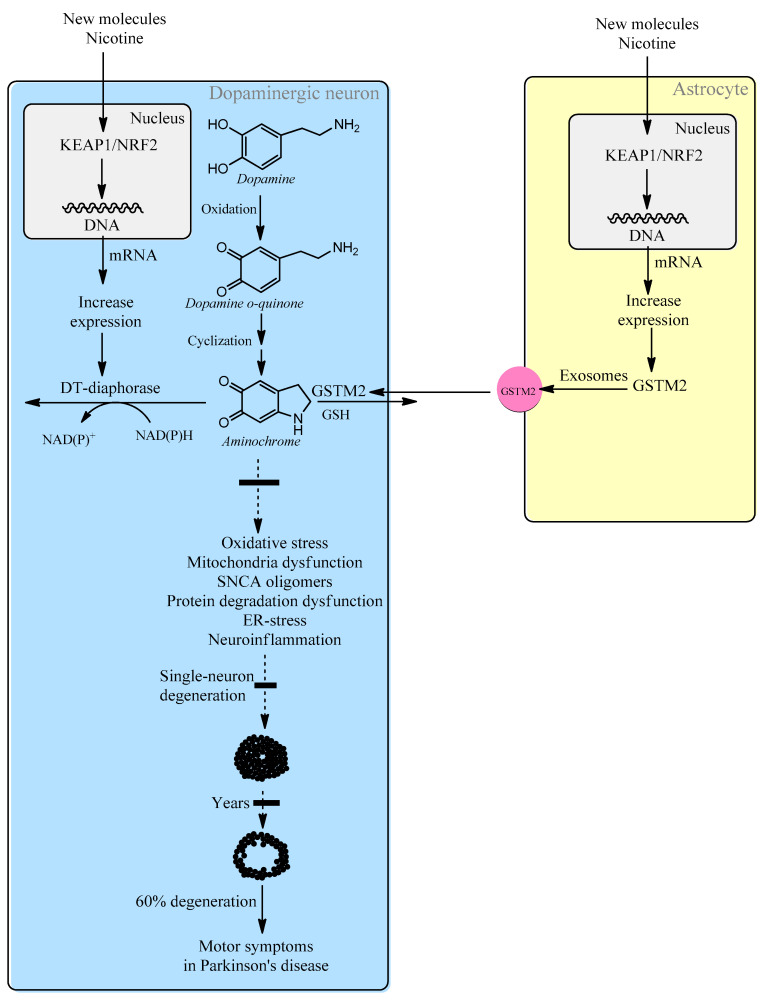
Intracellular increased expression of DT-diaphorase and glutathione transferase M2-2 through activation of the KEAP1/NRF2 signaling pathway will prevent single-neuron degeneration in idiopathic Parkinson’s disease.

In the single-neuron degeneration model, the endogenous neurotoxin aminochrome is the molecule that triggers the degenerative process that leads to the loss of neuromelanin-containing dopaminergic neurons in an individual neuron. However, aminochrome cannot be used in a preclinical animal model because it is technically impossible to inject a single neuron with aminochrome. An intracerebral injection will have a massive effect on all the neurons as far as the aminochrome injection reaches. Therefore, it is technically impossible to test new molecules for the treatment of Parkinson’s disease in a single-neuron degeneration model. For this reason, a new strategy and target for the treatment of idiopathic Parkinson’s disease is to search for molecules that activate the KEAP1/NRF2 signaling pathway that leads to the induction of increased expression of DT-diaphorase and glutathione transferase M2-2 (Figure 2). Molecules such as nicotine that activate this pathway and also inhibit the neurotoxic effects of aminochrome in cell cultures may be potential new drugs for the treatment of idiopathic Parkinson’s disease [138,139] (Table 7).

## 8. Conclusions

The absence of drugs that can halt or significantly slow the progression of idiopathic Parkinson’s disease requires the scientific community to explore new ideas such as the single-neuron degeneration model. This model of single-neuron neurodegeneration is based on the fact that the synthesis of neuromelanin can generate the endogenous neurotoxin aminochrome under certain circumstances. Neuromelanin synthesis is a normal and harmless process since healthy elderly people have neuromelanin-containing dopaminergic neurons intact in the substantia nigra at the time of death. However, the excessive production of aminochrome overcomes the neuroprotective capacity of the enzymes DT-diaphorase and glutathione transferase M2-2 that finally generates aminochrome neurotoxicity. The chemical characteristics of aminochrome, such as short stability time in the cytosol that depends on the presence of flavoenzymes that can reduce it or proteins with which it forms adducts, prevent it from having an expansive character that affects neighboring neurons [62,66,88,89]. This implies that aminochrome-induced neurotoxicity affects individual dopaminergic neurons, explaining the extremely slow rate of the degenerative process and progression of idiopathic Parkinson’s disease, which takes years.

If we agree that the oxidation of dopamine to aminochrome plays an essential role in the loss of neuromelanin-containing dopaminergic neurons in idiopathic Parkinson’s disease, we have to look for molecules that increase the expression of the enzymes DT-diaphorase and glutathione transferase M2-2 that prevent the neurotoxic effects of aminochrome, or molecules that inhibit the oxidation of dopamine to neuromelanin. The targets that our research should aim at in the search for new drugs for the treatment of idiopathic Parkinson’s disease include: (i) searching for molecules such as pramipexole that increase the expression of the VMAT2 transporter that prevents the existence of free dopamine that can oxidize the endogenous neurotoxin aminochrome during neuromelanin synthesis. Neuromelanin synthesis is inversely proportional to the level of VMAT2 expression. A higher expression of VMAT2 results in lower neuromelanin synthesis [106,107]; and (ii) searching for molecules that activate the KEAP1/NRF2 pathway to induce the expression of the neuroprotective enzymes DT-diaphorase and glutathione transferase M2-2 that prevent the neurotoxic effects of aminochrome during the synthesis of neuromelanin in dopaminergic neurons of the nigrostriatal system [78].

## Figures and Tables

**Table 1:** Summary.

Despite great advances in relating mechanisms related to the degenerative process in Parkinson’s disease, it is not known what triggers all of these mechanisms.
The discovery of genes associated with familial Parkinson’s disease has been a great contribution to basic research, but familial Parkinson’s disease represents only 5–10% of all Parkinson’s patients.
Many researchers believe that environmental factors play a relevant role in Parkinson’s disease. However, environmental factors such as manganese, copper, and paraquat induce Parkinsonism in young subjects. MPTP induces severe Parkinsonism after just three days of exposure.
Premotor symptoms have been associated with deposits of alpha-synuclein in Lewy bodies that expand from one region to another region of the brain, which have served as the basis for proposing different stages of the disease. However, it has been observed that the Braak stages are not valid for patients with late onset such as patients with idiopathic Parkinson’s disease.
It has been proposed that alpha-synuclein oligomers play a fundamental role in the loss of neuromelanin-containing dopaminergic neurons. However, the expansive nature of alpha-synuclein is the opposite of the extremely slow progression of the disease.
Aminochrome is an endogenous neurotoxin that induces all the mechanisms related to the neurodegenerative process of Parkinson’s disease.

**Table 2:** Summary.

Dopamine accumulates in monoaminergic vesicles where it is completely stable due to a slightly low pH that prevents its autoxidation.
Free dopamine in the cytosol can be oxidized to form neuromelanin through the formation of ortho-quinones such as aminochrome that are potentially neurotoxic.
Aminochrome is a transient metabolite that is formed during neuromelanin synthesis and does not induce a propagative neurotoxic effect towards neighboring neu-rons.

**Table 3:** Summary.

Neuromelanin synthesis can be a normal and harmless process that requires the formation of the endogenous neurotoxin aminochrome due to the existence of DT-diaphorase and glutathione transferase M2-2 prevent the neuro-toxic effects of aminochrome.
Astrocytes secrete glutathione transferase M2-2 through exosomes that penetrate dopaminergic neurons, releasing this enzyme inside the cytosol of these neurons.

**Table 4:** Summary.

VMAT-2, which transports dopamine into monoaminergic vesicles, forms a kind of complex with tyrosine hydroxylase and aromatic enzyme L-amino acid decarboxylase that prevents the existence of free dopamine during dopamine synthesis.
VMAT-2 forms a kind of complex with the dopamine transporter and syn-aptogyrin-3 that prevent the existence of free dopamine in the cytosol during its reuptake after neurotransmission.
There is an inverse relationship between neuromelanin levels and VMAT-2 ex-pression

**Table 5:** Summary.

There is a long list of failed clinical studies that have been based on preclinical models with exogenous neurotoxins.
A possible explanation for these failures is that preclinical models based on exogenous neurotoxins do not represent what happens in the neurodegenerative process of the disease.
The degenerative process of dopaminergic neurons that contain neuro-melanin is an extremely slow process that takes years where the therapeutic effects of drugs that have failed in clinical studies were measured in very rapid and expansive preclinical models cannot be observed.

**Table 6:** Summary.

The essential role of VMAT2 in preventing dopamine oxidation suggests that its overexpression may be a therapeutic target to develop new drugs that slow the progression of the disease.
Pramipexole is a dopamine agonist used in the therapy of Parkinson’s dis-ease and SPECT studies have demonstrated a neuroprotective effect in patients with Parkin-son’s disease.
Pramipexole induces the expression of VMAT2 mRNA levels

**Table 7:** Summary.

It has been proposed that the single-neuron degeneration model where the de-generative process of idiopathic Parkinson’s disease affects a single neuron individually without expansive effects, where the neurotoxin that triggers this degenerative process is generated within these neurons. affected and does not have expansive effects.
Aminochrome is a good candidate for this single-neuron degeneration model because (i) it is formed within neuromelanin-containing dopaminergic neurons; (ii) it does not have expansive effects towards neighboring neurons; (iii) induces mitochondrial dysfunction and protein degradation systems, oxidative stress, formation of neurotoxic alpha-synuclein oligomers, endoplasmic reticulum stress and neuroinflammation.
Aminochrome cannot be used in a preclinical animal model because it is technically impossible to inject a single neuron with aminochrome. An in-tracerebral injection will have a massive effect on all the neurons as far as the aminochrome injection reaches.
A new strategy to search for new drugs for the treatment of idiopathic Park-inson’s disease is to search for molecules that activate KEAP1/NRF2 signal-ing pathway that leads to the induction of increased expression of DT-diaphorase and glutathione transferase M2-2.
